# Targeted volume correlative light and electron microscopy of an environmental marine microorganism

**DOI:** 10.1242/jcs.261355

**Published:** 2023-08-09

**Authors:** Karel Mocaer, Giulia Mizzon, Manuel Gunkel, Aliaksandr Halavatyi, Anna Steyer, Viola Oorschot, Martin Schorb, Charlotte Le Kieffre, Daniel P. Yee, Fabien Chevalier, Benoit Gallet, Johan Decelle, Yannick Schwab, Paolo Ronchi

**Affiliations:** ^1^ Cell Biology and Biophysics Unit, European Molecular Biology Laboratory, 69117 Heidelberg, Germany; ^2^ Collaboration for joint PhD degree between the European Molecular Biology Laboratory and the Heidelberg University, Faculty of Biosciences, 69120 Heidelberg, Germany; ^3^ Electron Microscopy Core Facility, European Molecular Biology Laboratory, 69117 Heidelberg, Germany; ^4^ Department of Infectious Diseases, Molecular Virology, CIID, 69120 Heidelberg, Germany; ^5^ German Center for Infection Research, Heidelberg partner site, 69120 Heidelberg, Germany; ^6^ Advanced Light Microscopy Facility, European Molecular Biology Laboratory, 69117 Heidelberg, Germany; ^7^ EMBL Imaging Centre, European Molecular Biology Laboratory, 69117 Heidelberg, Germany; ^8^ Université Grenoble Alpes, CNRS, CEA, INRAe, IRIG-LPCV, 38054 Grenoble, France; ^9^ Institut de Biologie Structurale (IBS), Université Grenoble Alpes, CEA, CNRS, 38000 Grenoble, France

**Keywords:** Focused ion beam-scanning electron microscopy, Correlative light and electron microscopy, Volume electron microscopy, Environmental sample, Plankton, Dinoflagellate

## Abstract

Photosynthetic microalgae are responsible for an important fraction of CO_2_ fixation and O_2_ production on Earth*.* Three-dimensional (3D) ultrastructural characterization of these organisms in their natural environment can contribute to a deeper understanding of their cell biology. However, the low throughput of volume electron microscopy (vEM) methods along with the complexity and heterogeneity of environmental samples pose great technical challenges. In the present study, we used a workflow based on a specific electron microscopy sample preparation method compatible with both light and vEM imaging in order to target one cell among a complex natural community. This method revealed the 3D subcellular landscape of a photosynthetic dinoflagellate, which we identified as *Ensiculifera tyrrhenica*, with quantitative characterization of multiple organelles. We show that this cell contains a single convoluted chloroplast and show the arrangement of the flagellar apparatus with its associated photosensitive elements. Moreover, we observed partial chromatin unfolding, potentially associated with transcription activity in these organisms, in which chromosomes are permanently condensed. Together with providing insights in dinoflagellate biology, this proof-of-principle study illustrates an efficient tool for the targeted ultrastructural analysis of environmental microorganisms in heterogeneous mixes.

## Teaser

**Highlighted Article:** We developed a correlative light and electron microscopy workflow to target environmental microorganisms from heterogeneous samples and, as proof of principle, revealed the 3D ultrastructure of a marine dinoflagellate.

## INTRODUCTION

Electron microscopy (EM) has played an essential role in understanding cell biology by revealing the intracellular organization in a vast variety of cells, tissues and small organisms (McIntosh, 2007). More recently, volume EM (vEM) methods have made it possible to visualize ultrastructure in three dimensions (3D) (Peddie et al., 2022), opening the way to new discoveries. However, to date, these methods have been rarely applied to study marine microplankton. Microplankton are microorganisms that populate aquatic ecosystems. They include a wide variety of species ranging from prokaryotes to eukaryotic microalgae. Our understanding of their cell biology is still very limited. Indeed, working with highly heterogeneous environmental samples is very challenging, and only a small fraction of species can be cultured in the laboratory (Dixon and Syrett, 1988; Oliveira et al., 2020). Therefore, new methods to characterize these cells in their native ecosystem is highly needed.

In this study, we present a workflow to characterize microorganisms from environmental samples by vEM, addressing the bottlenecks discussed previously. For this proof of principle, we focused on a dinoflagellate cell. Dinoflagellates represent a considerable fraction of plankton and play an important role in the aquatic food chain (De Vargas et al., 2015). There are close to 2400 described species, which are highly heterogeneous in morphology, trophic mode and distribution (Gómez, 2012). Approximately half of them are primary producers contributing to O_2_ production and CO_2_ fixation on the planet (Gómez, 2012). Similar to most marine planktonic cells, dinoflagellates are difficult to maintain in culture (Dixon and Syrett, 1988). Therefore, only a small fraction of species has been thoroughly investigated. Nonetheless, a coarse picture of the subcellular characteristics of dinoflagellates can be extracted from past EM studies conducted on cultured species.

One of the most striking features of these organisms is that they have numerous (up to 200) chromosomes (Bhaud et al., 2000), which stay permanently condensed throughout their cell cycle (Gautier et al., 1986). A fraction of these organisms possesses rigid cellulose plates located in a single layer of flattened vesicles lying under the plasma membrane, forming the theca. This contributes to the distinctive shape of the cells and is often used for taxonomic classification. Characteristic organelles of the dinoflagellate are trichocysts, described as rod-shaped crystalline structures with a square cross-section profile (Bouck and Sweeney, 1966). These structures can be extruded from the cell (Westermann et al., 2015), potentially as a defense mechanism. However, their function is still highly debated (Plattner, 2017). Moreover, dinoflagellates generally present a Golgi complex hemispherically distributed above the nucleus (Dodge, 1971), as well as secretory organelles of various shapes and content known as mucocysts (Hoppenrath, 2017). Dinoflagellates generally present two flagella, important for cellular movement (Dodge, 1971). Closely associated to the flagella, these organisms can have photosensitive structures called eyespots, potentially responsible for directionality of their movement (Dodge, 1984). As the picture described above is derived from transmission EM (TEM) studies, with a few exceptions where vEM was used (Decelle et al., 2021, 2022; Gavelis et al., 2019; Uwizeye et al., 2021a,b), the 3D understanding of their organization is still largely lacking. Importantly, it has been described that some cells can lose specific structures, for instance, the eyespot, when kept in culture (Moldrup et al., 2013). Therefore, implementing culture-independent methods to study planktonic cells in their native ecosystem is truly important to better understand the cellular biology of these ecologically relevant microorganisms.

Here, we present a workflow that enables the identification of a microalgal taxon of interest in a highly heterogenous environmental sample, containing hundreds of cells of diverse species. We used an EM sample preparation method that allows correlative analysis of the 3D cell fluorescence pattern and focused ion beam-scanning EM (FIB-SEM) acquisition. To this aim, we further optimized the workflow presented by Ronchi et al. (2021). This enabled us to efficiently generate a vEM dataset of a photosynthetic dinoflagellate from a complex environmental community. The analysis of the 3D ultrastructure allowed us to understand the intracellular organization of the organelles, revealing new insight in the biology of this microorganism.

## RESULTS

### Workflow

**Fig. 1.**
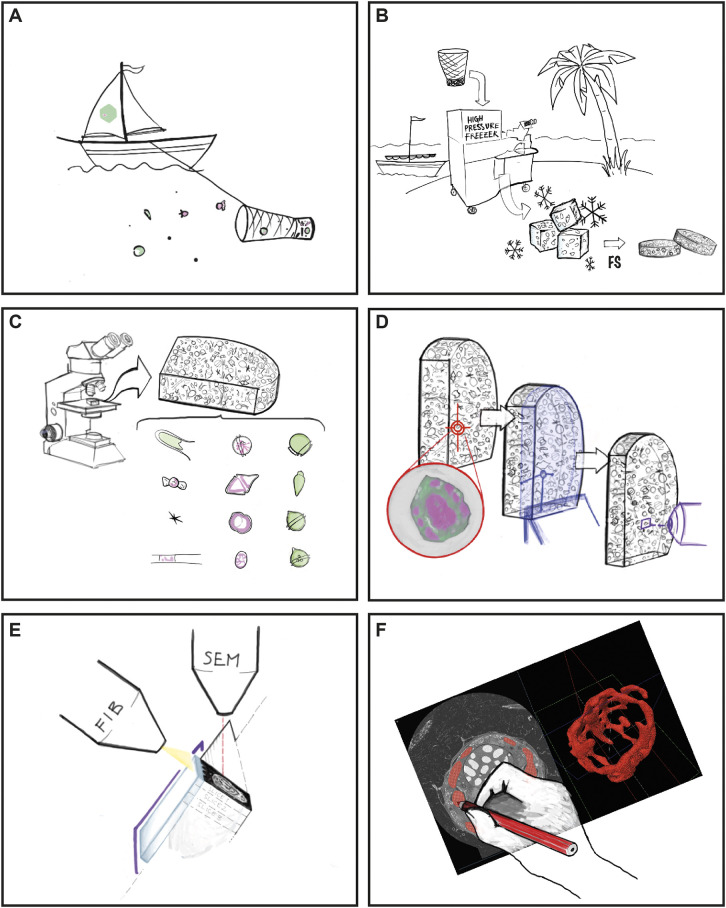
**Workflow of the study.** (A) Sample collection in the Villefranche-sur-Mer bay area, Mediterranean sea, France. (B) High-pressure freezing in close proximity to the sampling site, later followed by freeze substitution (FS) and resin embedding. (C) Mapping of the block using confocal microscopy and identification of various microorganisms present in the heterogeneous sample. (D) Targeting of a specific cell and determination of its *x*, *y* and *z* coordinates (red target), followed by ultramicrotome trimming to approach the cell of interest in *z* (in blue) and finally laser branding to mark the cell position in *x* and *y* on the block surface (in purple). (E) FIB-SEM acquisition of the cell of interest. (F) Segmentation of the organelles of the targeted cell. Here, the chloroplast is shown during segmentation and after rendering in red.

In order to study environmental marine microorganisms, we established a new workflow (Fig. 1). It consists of collection at sea (Fig. 1A), fractionation and concentration of the sample, followed by its rapid cryo-immobilization on site (Fig. 1B, see Materials and Methods). These frozen samples are then freeze substituted and resin embedded according to Ronchi et al. (2021), in order to preserve the autofluorescence properties of the cells (Fig. 1B). A 3D map of the entire block is generated by confocal microscopy, allowing us to identify a variety of microorganisms and their genus (Fig. 1C). A specific cell of interest can then be localized in *x* and *y* and in relation to the block's surface (Fig. 1D). After trimming away the resin on top of the targeted cell, branding of a pattern around this organism (Fig. 1D) is performed to allow for its precise acquisition using FIB-SEM imaging (Fig. 1E). The dataset is then aligned and semi-automated segmentation is performed for further analysis of lengths and volumes of various subcellular structures, as well as their visualization (Fig. 1F). The individual steps of this workflow are further detailed below.

### Sample preparation and cell identification

Preparation of marine environmental samples for ultrastructural studies by EM is highly complex. The quality of preservation is very time sensitive as planktonic microorganisms are delicate and susceptible to distortions before and during fixation (Truby, 1997). To help overcome these challenges, the samples used in this study were cryo-immobilized by high-pressure freezing within 2 h after collection in a custom set up implemented in a marine station (see Materials and Methods).

**Fig. 2.**
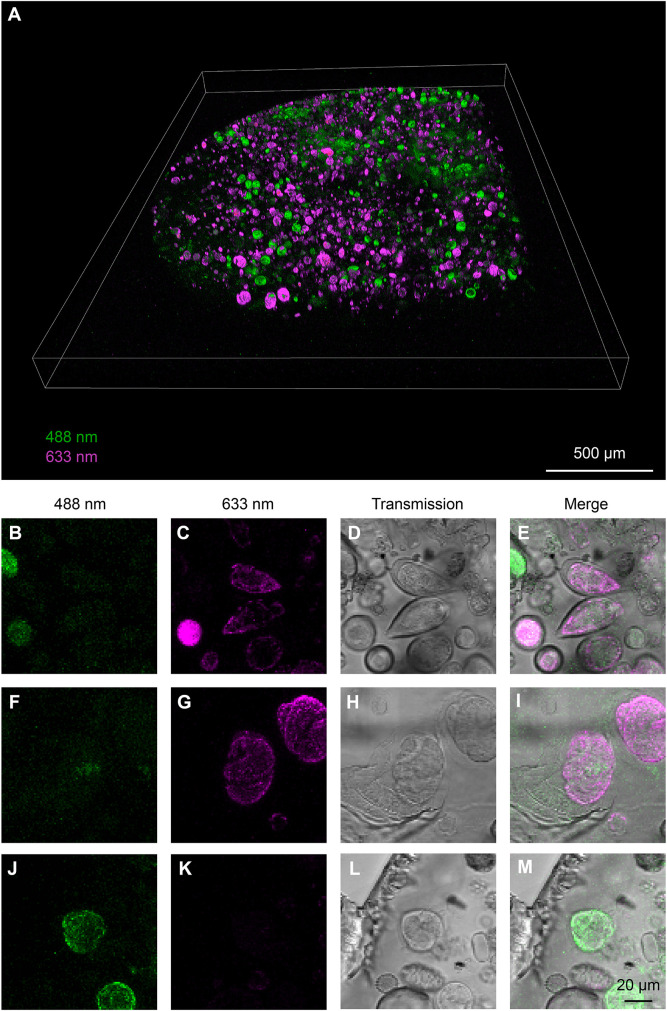
**Confocal characterization of the high-pressure-frozen freeze-substituted planktonic sample embedded in a plastic block.** (A) 3D rendering of the two-color tiled *z*-stack confocal acquisition of the resin block. (B–M) Fluorescence (B,C,F,G,J,M) and transmitted light (D,H,L) imaging of three different cells from A. The imaging settings are the same for the different cells in each channel. Maximum-intensity projections of the confocal stacks are displayed for both fluorescence channels. For the transmitted light channel, single slices are shown. Fluorescence and transmitted light images are overlaid in E,I,M. The cells were putatively identified as belonging to the genera *Prorocentrum* (B–E), *Cochlodinium* (F–I) and *Protoperidinium* (J–M).

Each frozen sample contained hundreds of cells representing diverse plankton taxa. As most microalgae display characteristic autofluorescence properties, we decided to use light microscopy to target specific cells of interest. We therefore performed a freeze-substitution method for preserving fluorescence by using a low amount of heavy metals and embedding in Lowicryl HM20 (Kukulski et al., 2011; Nixon et al., 2009; Porrati et al., 2019; Ronchi et al., 2021). Confocal 3D imaging of the resulting block revealed that the autofluorescence pattern was preserved after sample preparation and could be detected in the block for the entire thickness of the high-pressure frozen material (200 µm) (Fig. 2A; Movie 1). Using excitation light at 488 and 633 nm along with transmitted light to visualize cell morphology, we could distinguish different genera in the block. For instance, we could observe dinoflagellates such as *Protoperidinium*, *Prorocentrum* or *Oxytoxum*, as well as other organisms such as diatoms or coccolithophores (Fig. 2B–M; Fig. S1). We could also identify damaged cells to exclude from downstream processing. As chlorophyll *a* has been reported to be autofluorescent in the far red (Hense et al., 2008), we expected to be able to discriminate between photosynthetic and non-photosynthetic organisms by the presence of an emitted signal upon illumination at 633 nm.

**Fig. 3.**
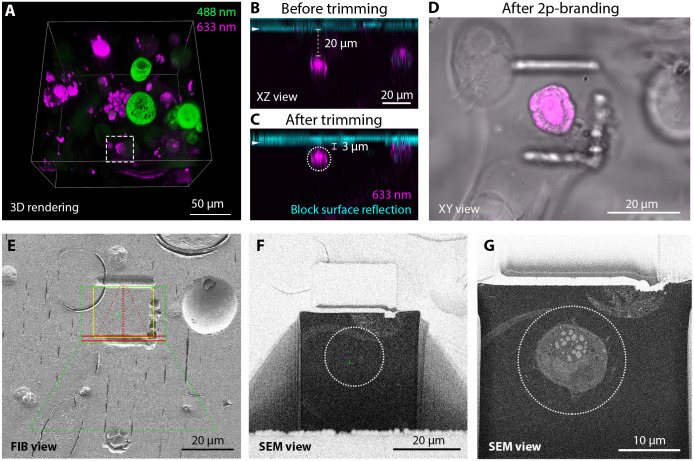
**Targeting of a cell of interest (a photosynthetic dinoflagellate).** (A) 3D rendering of a high-resolution confocal stack in a selected area allows the identification of the target dinoflagellate cell (dashed square). (B,C) Confocal *xz* views of the cell of interest in the resin block before (B) and after (C) the trimming steps. The arrowhead indicates the block surface, as visualized in the reflection channel (cyan). Distance between the upper edge of the cell and the block surface is displayed. The dashed circle corresponds to the target position of the cell to be acquired by FIB-SEM. (D) NIR branding of the block surface generates landmarks around the cell of interest, visualized by transmitted light. (E) The embossed lines generated by the branding are visible by FIB imaging. These lines are used to define the region to be acquired by FIB-SEM. The overlaid profiles (green trapezoid and rectangle, red lines and yellow bounding box) illustrate the software (Atlas) sample preparation shapes used to define the slice-and-view acquisition. (F) SEM view of the imaging surface after FIB sample preparation, right before starting the acquisition. The cell of interest is not exposed yet and the dashed circle represents its predicted position from Fig. 2C. (G) Low magnification SEM overview (keyframe) during the acquisition, showing the precision of the region-of-interest prediction. The dashed circle is in the same position as in F.

Considering the information provided by light microscopy, for this study, we used FIB-SEM for the vEM analysis of a plastid-bearing dinoflagellate. Therefore, using transmitted light, we selected a cell that presented a transverse groove and one longitudinal located antiapically (Movie 1), as is typical of many dinoflagellates. From the cells showing this particular shape, we further selected an organism that displayed a far-red signal when excited at 633 nm. The autofluorescence pattern consisted of a globular shape in the center of the cell and more defined patches at the cell periphery (Fig. 3A–D; Movie 1), which we hypothesized to be chloroplasts.

### Cell targeting and acquisition

In order to image the cell of interest by FIB-SEM, we optimized the strategy reported by Ronchi et al. (2021), based on a two-step targeting workflow. First, the distance of the cell from the block surface was measured from a confocal stack (Fig. 3A,B). To determine the exact position of the block surface, we used the reflection of the laser light at the interface between materials with different refractive indexes (the water used as a mounting medium and Lowicryl of the block; Fig. 3B, arrowhead). Then, we removed the measured thickness of resin present above the cell of interest with a trimming knife mounted on an ultramicrotome in three iterations of imaging and trimming. Once the cell was located just below the surface (Fig. 3C), we branded landmarks on the block surface using a near infrared (NIR, ‘two-photon’) laser in order to later facilitate its targeting by FIB-SEM (Fig. 3D). Indeed, such branded landmarks were easily identified by SEM and were used to define the FIB milling area (Fig. 3E). A trench was then opened to approach the cell according to its predicted location (Fig. 3F). Using the measurements of the confocal stack (Fig. 3C, dashed circle), we could precisely predict the position of the acquisition window where the cell would appear (Fig. 3F, dashed circle). The FIB-SEM automated acquisition was then started and the entire cell was acquired at 8 nm isotropic voxel size (Fig. 3G; Movie 2) [the complete dataset is available for download at the Electron Microscopy Public Image Archive (EMPIAR) under the accession number EMPIAR-11399]. The complete dataset and associated segmentation can be visualized and interacted with using MoBIE (https://github.com/mobie/environmental-dinoflagellate-vCLEM; see Materials and Methods; Movie 1). The quality of the data acquired was consistent with previously published volume correlative light and electron microscopy (CLEM) datasets (D'Imprima et al., 2023; Porrati et al., 2019; Ronchi et al., 2021).

**Fig. 4.**
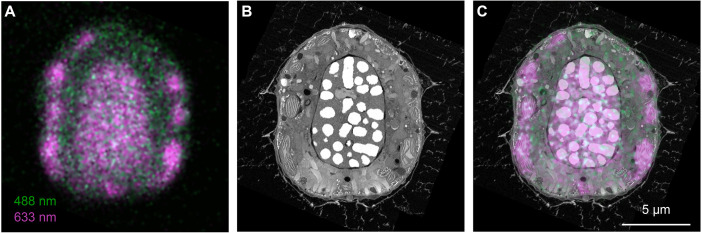
**Overlay of autofluorescence signal and ultrastructure from vEM.** (A) Fluorescence pattern of the cell of interest. The image shows a confocal slice along the longitudinal axis, with an optical thickness of 2.2 µm. (B) Single orthoslice through the FIB-SEM volume in the region corresponding to the fluorescence signal. (C) Fluorescence and FIB-SEM overlay. A single slice of the overlay is shown. The pattern of the 633 nm excited signal clearly overlaps with the position of the chloroplast and nucleus.

By overlaying the light microscopy and the FIB-SEM data, we tried to confirm the nature of the autofluorescence signal. We observed that the far-red signal matched the position of the chloroplast and the nucleus (Fig. 4A–C; Movie 1). Although we cannot explain the autofluorescence in the nucleus, the overlap with the plastid is justified by the presence of chlorophyll (Hense et al., 2008). This confirms that the far-red signal from microorganisms within the block can be used for the identification of photosynthetic species. Additionally, although it has not been explained, autofluorescence signals such as the one emitted here by the nuclear region can contribute to a finer discrimination between cell types.

### Ultrastructural characterization

**Fig. 5.**
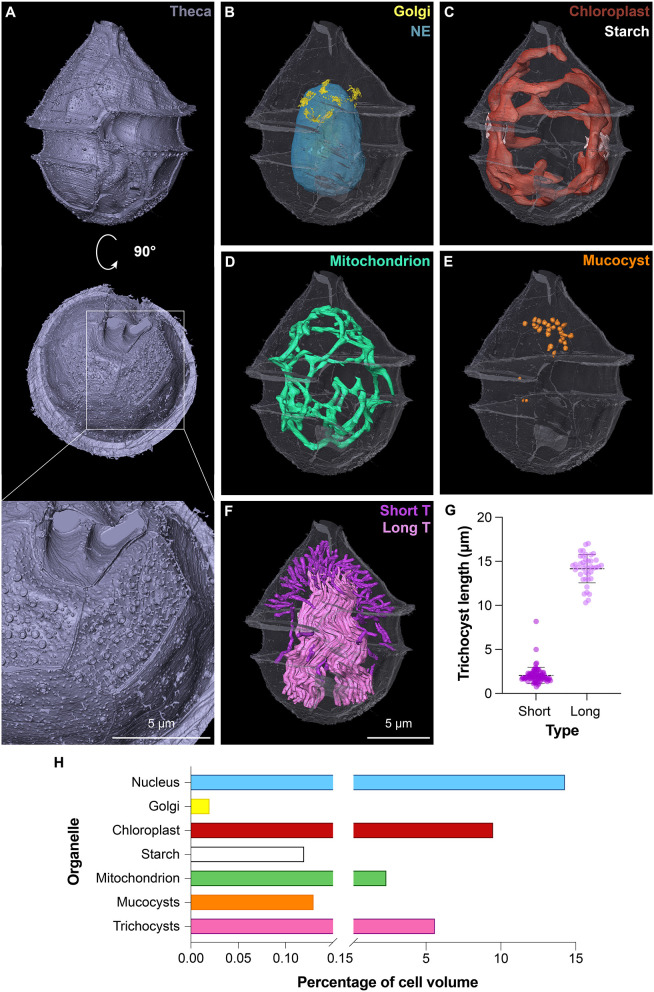
**Morphometrics of organelles in the targeted photosynthetic dinoflagellate.** (A) 3D rendering of the theca in ventral (top) and antiapical views (center, bottom). The enlargement of the antiapical view in the lower panel shows the pore arrangement and presence of small knobs on the lower plates. All images of intracellular organelle segmentation (B–E) are shown in the same orientation as the top panel (ventral view), with the theca shown in transparency. (B) Segmentation of the nucleus/nuclear envelope (NE) (cyan) and Golgi apparatus (yellow). (C) Segmentation of the single convoluted chloroplast (red) and associated starch (white). (D) Segmentation of the mitochondrion (green). (E) Segmentation of the mucocysts (orange). (F) Segmentation of the trichocysts (T). Short linear trichocysts are shown in magenta. Long and convoluted trichocysts are in light pink. (G) Size distribution of the two classes of trichocysts. *n*=80 for the short and 41 for the long class. Each individual point represents the measurement of a trichocyst. Means±s.d. are shown on the graph. (H) Volumes of the segmented organelles expressed as relative percentage of the full cell volume (1008.76 µm^3^).

We then segmented a set of characteristic organelles of the cell from the FIB-SEM stack (Fig. 5; Movie 2) and performed subsequent morphometric analysis. The cell measured 16 µm in height and 13.5 µm in width, for a total volume of 1009 µm^3^. It showed a conical epitheca as well as a wide and deep cingular girdle. The cell surface was well preserved, allowing most thecal plates to be counted and described individually (with the exception of the sulcal plates). Elucidation of the plate tabulation was important for taxonomical identification. Traditionally, this analysis is done using SEM; however, we show here that it can be also obtained from the reconstruction of the FIB-SEM volume. From our analysis, we could observe the following thecal arrangement according to the Kofoidian system (Fensome, 1993): x, 4′, 3a, 7″, 4c+T, 5‴, 2″″. The topography of the different thecal plates displayed circular pores surrounded by small knobs or bumps (Fig. 5A). The pores were either linearly arranged as, for instance, above and under the cingulum or distributed with various densities throughout a given plate (Fig. 5A, enlarged inset). Small knobs were also distributed unevenly throughout the thecal plates. Given the size range, outer morphology and the tabulation, we identified this organism as *Ensiculifera tyrrhenica* [synonym to *Pentapharsodinium tyrrhenicum* (Balech)]*,* a dinoflagellate belonging to the class Dinophyceae and order Peridiniales. Analysis of the environmental sample collected in parallel and processed for topography SEM confirmed the presence of this species (Fig. S2). Interestingly, the position of the thecal openings and details of the ornamentation of the organisms analyzed with the two methods were extremely similar (Fig. S2), validating our FIB-SEM imaging-based analysis as a tool for determining taxonomic features. However, vEM has the additional advantage to provide insights on the intracellular morphology of the cell, which cannot be appreciated with SEM alone.

Segmentation of some intracellular organelles allowed us to assess their position as well as size, shape and volume (Fig. 5; Movie 2). The nucleus, representing 14.3% of the volume of the cell, had an elliptical shape and was located centrally in the posterior part of the cell, with its major axis aligned with the long axis of the cell (Fig. 5B,H). The Golgi apparatus was organized in 12 stacks located in the ventral apical region of the cell close to the nucleus (Fig. 5B; Fig. S3A,B), and occupied 0.02% of the cell volume (Fig. 5H).

Classical taxonomical description of the genus *Ensiculifera* based on light microscopy reported the presence of reticulated chloroplasts (Li et al., 2020). However, identification of the number of chloroplasts or the pyrenoid distribution is difficult to interpret from light microscopy or even TEM images. Our vEM analysis allowed us to unambiguously resolve the 3D organization of this organelle as a single convoluted and interconnected structure (Fig. 5C). The chloroplast represented 9.5% of the total cell volume (Fig. 5H). From the raw data, we could also appreciate the organization of the thylakoids and pyrenoid (Fig. S3C,D). Associated to the chloroplast, we observed the presence of starch, representing 0.12% of the cell volume and confined around two opposite lobes of the plastid where the pyrenoid was located (Fig. 5C,H; Fig. S3C,D). Of note, the sample was collected before sunrise, and a low amount of starch is compatible with night starch consumption (Seo and Fritz, 2002).

In close proximity to the inner side of the chloroplast, we visualized an intricate mitochondrial network representing 2.37% of the cell volume (Fig. 5D,H; Movie 2). As observed for the chloroplast, the mitochondrion consisted of a single interconnected structure. Interestingly, the two organelles were closely associated throughout the cell volume (Movie 2), as observed in other microalgae (Uwizeye et al., 2021b).

We further analyzed the trichocysts, organelles typically found in dinoflagellates and described as rod-shaped crystalline structures originating from the Golgi area with a square profile when cut transversely (Bouck and Sweeney, 1966). Our 3D analysis confirmed these general features of the trichocysts (Fig. S3E,F), but further allowed us to divide them in two classes based on their length and distribution (Fig. 5F,G). One class consisted of short (2.04±0.92 µm, indicated as mean±s.d., Fig. 5G) and straight structures, often perpendicular to the plasma membrane in the apical region (Fig. 5F, magenta). The second class formed a bundle of long (14.17±1.61 µm, Fig. 5G), twisted and intricated trichocysts stretching along the longer cellular axis (Fig. 5F, light pink). Contrary to our expectations, neither class aligned with the thecal circular openings. Altogether, trichocysts occupied a significant fraction of the cell volume (5.61%, Fig. 5H). We further analyzed secretory organelles, described as mucocysts in dinoflagellates. We identified 30 amphora-shaped structures (Fig. S3G,H), with an average volume of 0.046±0.017 µm^3^. Altogether, mucocysts occupied 0.13% of the cell volume and were clustered under the plasma membrane in the posterior apical region of the cell (Fig. 5E,H).

**Fig. 6.**
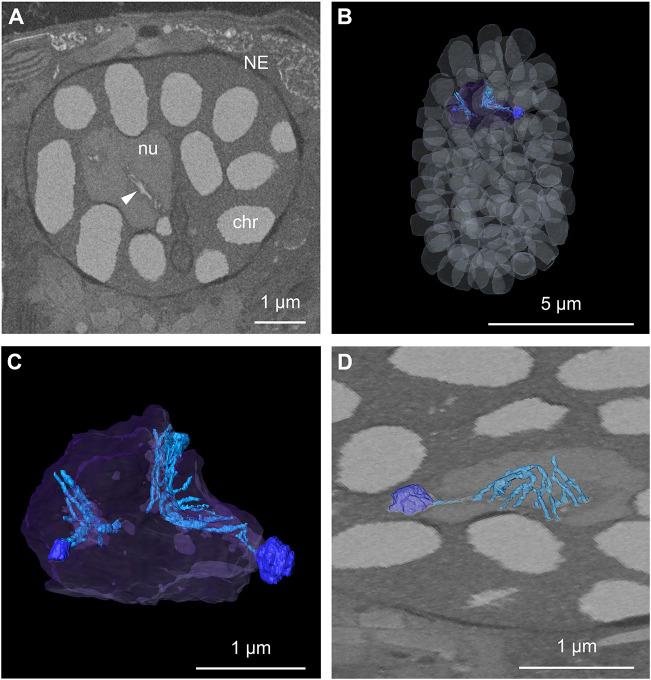
**Nucleus and chromatin organization.** (A) Single orthoslice through the FIB-SEM volume in the nuclear region (NE, nuclear envelope; nu, nucleolus, chr, condensed chromosome). The arrowhead highlights filamentous structures originating from a small chromosome and expanding in the nucleolus. (B) 3D rendering of the segmentation of the chromatin (white in transparency), nucleolus (purple in transparency) and filamentous structure (light blue) associated with small chromosomes located adjacent to the nucleolus (dark blue). (C) Close up view of the segmentation of nucleolus (purple in transparency) with associated small chromosomes (dark blue) and extended filamentous structure (light blue). (D) Rendering of the segmentation of the intranucleolar filamentous chromatin structure overlaid with an image of the raw data, illustrating the connection between the filament and the small chromosome associated with the nucleolus.

Next, we performed a detailed characterization of the nuclear organization (Fig. 6). Chromatin segmentation revealed that the nucleus contained 105 condensed chromosomes (Fig. 6A,B). Their volume was on average 0.510±0.162 µm^3^. Interestingly we could also observe two chromosomes located adjacent to the nucleolus that appeared smaller (0.025 µm^3^ and 0.004 µm^3^) compared to the other chromosomes. A detailed analysis of these structures showed threads of electron-dense material originating from the chromosomes and extending within the nucleolar space in a convoluted manner (Fig. 6C,D). The electron-density properties, similar to those of neighboring chromosomes (Fig. 6A, arrowhead), suggested that these filamentous structures could be chromatin in an intermediate compaction state.

**Fig. 7.**
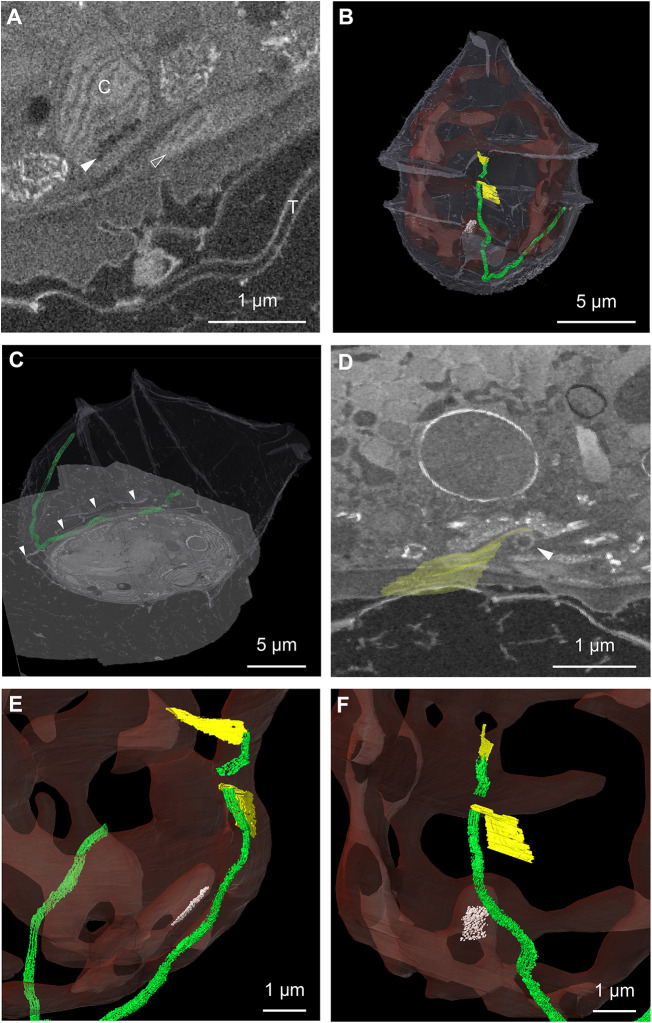
**3D organization of the flagellar apparatus and eyespot.** (A) Single orthoslice through the FIB-SEM volume in the eyespot region (C, chloroplast; T, theca). The filled arrowhead indicates the eyespot and empty arrowhead indicates the longitudinal flagellum. (B) 3D rendering of the segmentation of the eyespot (white), located within the chloroplast (red in transparency), and of the flagella (green) and associated filaments (yellow). The theca is shown in white in transparency. (C) Rendering of the segmentation of the longitudinal flagellum overlaid with a slice of the FIB-SEM volume. Arrowheads indicate the position of the theca, demonstrating that the flagellum extends inside the theca. (D) Rendering of the segmentation of a microtubule sheet (yellow) overlaid with a slice through the volume, showing the basal body of the flagellum (arrowhead). (E,F) Close up of the segmentation of the chloroplast (red in transparency), flagella (green) and associated filaments (yellow), as well as the eyespot (white) located within the chloroplast. Part of the array, close to the longitudinal flagellum, could not be fully discriminated with the resolution of our dataset. Even though the array seemed to extend towards the eyespot, segmentation was performed only on the part we could unambiguously assign.

Furthermore, we looked at flagella, characteristic structures of dinoflagellates, and the associated eyespot (Fig. 7A), a putative photosensitive structure (Colley and Nilsson, 2016). The cell displayed two flagella, protruding from basal bodies located underneath the intersection between the sulcus and cingulum (Fig. 7B). These structures were elongated in the space between the plasma membrane and theca (Fig. 7C), a position that, to our knowledge, had not been described previously. The longitudinal flagellum appeared very long and wrapped half of the cell perimeter, whereas the transverse flagellum was very short, and might have been affected during sample collection. The eyespot could be visualized behind the sulcus groove (Fig. 7B), as reported for other Peridiniales (Dodge, 1984; Li et al., 2020). The eyespot here consisted of a single layer of globules within the chloroplast and was localized parallel to the longitudinal flagellum (Fig. 7E,F). Based on this arrangement, we believe it belongs to category I(A) of the eyespot classification (Hoppenrath, 2017), which has previously been described for other organisms from the family of Peridiniaceae (Calado et al., 1999; Messer and Ben-Shaul, 1969; Moestrup and Daugbjerg, 2007). For many dinoflagellate species, the presence of flat arrays of laterally connected microtubules in the basal body area have been reported (Calado and Moestrup, 2002; Calado et al., 1999), which were suggested to play a role in light-dependent movements (Dodge, 1984). Although the size of microtubules is at the limit of what can be resolved using our FIB-SEM imaging settings, we were able to visualize two structures resembling filamentous arrays, each one closely associated to a basal body (Fig. 7D–F). Taking advantage of the high density provided by the bundling of microtubules, we were able to localize them and determine their spatial arrangement. Although the filaments related to the basal body of the longitudinal flagellum were directed toward the cell surface, following the curvature of the plasma membrane, the filaments related to the basal body of the transverse flagellum were directed towards the inner part of the cell (Fig. 7E,F).

## DISCUSSION

In this study, we present a 3D CLEM workflow for the identification and ultrastructural analysis of single organisms from a very heterogeneous environmental marine sample.

When using vEM, it is often necessary to limit the acquisition to a few ‘representative’ individuals in a population because of the intrinsic low throughput of the method. In contrast to laboratory monocultures where inter-individual variability is usually relatively low, environmental samples can contain hundreds of species. Therefore, one of the biggest hurdles of meaningful 3D ultrastructural analysis of field samples is the identification and targeting of specific microorganisms. Using fluorescence profiling within the block, our method provides a novel way to explore heterogeneous samples to identify and select candidates for vEM acquisition. A similar workflow has been shown to work for exogenously expressed fluorescent proteins (Ronchi et al., 2021) or small-molecule live dyes (D'Imprima et al., 2023), with a success rate close to 100%. We have now demonstrated that the same principle can be applied to endogenous fluorescence signals with similar robustness, opening the way for environmental sample analysis. From the shape and fluorescence pattern observed in the confocal stack, we were able to target a plastid-bearing unicellular armored dinoflagellate. Following FIB-SEM imaging, we could confirm our prediction and identify the species by the thecal organization as *E. tyrrhenica.*

Another advantage that comes with the precision of the workflow is the reduction of the acquired FIB-SEM volume. Indeed, while a low-precision targeting would lead to the acquisition of a large buffer volume around the region of interest, with this workflow, we were able to restrict the acquisition very precisely around the cell of interest. This allowed us to optimize the imaging time and generate the entire dataset of the cell (∼15 µm diameter) in less than 48 h. However, depending on the targeted volume, the time for imaging will vary. For instance, datasets for some of the larger cells found in our samples (around 40 µm in diameter) would be acquired in 5 days using the same imaging settings. In the future, using this dataset as a reference and taking advantage of specific autofluorescence signatures, it might be possible to further restrict the vEM acquisition to a subcellular volume to answer specific biological questions. Overall, such an optimization of the acquisition time can then allow a scale up of vEM analyses. Segmentation is another crucial but time-consuming part of the workflow. However, with experience, the segmentation can be significantly accelerated. The almost entirely manual approach we used for this study (see Materials and Methods) took a trained user approximately 72 h. We envision that artificial intelligence can contribute towards automating this step, therefore removing another bottleneck towards scaling up of the method (Heinrich et al., 2021).

Our study is not only a methodological proof of concept but represents one of the first examples of vEM on environmental samples. More specifically, we describe here the subcellular organization of *E. tyrrhenica* for the first time. As datasets of this sort are rare and precious for the community, we believe that it is important to make them available. Thus, the raw dataset as well as the segmentations described in this study are accessible in EMPIAR (accession ID EMPIAR-11399) as well as in an easily browsable format using Fiji through the Mobie plugin (Pape et al., 2023).

With this vEM dataset, we could visualize the positioning of various structures that showed that the cell is highly polarized. Indeed, a subset of organelles are particularly concentrated in the apical region of the cell, such as the Golgi apparatus and mucocysts. The distribution of the trichocysts, particularly the short ones, appears polarized as well. They radiate from the Golgi area and are directed towards the apical plasma membrane, suggesting that they could be mature and ready for extrusion. Furthermore, the 3D analysis allowed us to observe the position of subcellular structures relative to one another as for the eyespot, flagella and associated filaments, which would be very difficult using other methods. The close association of the arrays of filaments with each basal body suggests that these microtubules could play a role in orienting the movement of the flagella. A higher-resolution imaging of this area could further reveal whether these arrays are associated with the eyespot, which might in this way determine the directionality of the movement as suggested by Dodge (1984).

The permanently condensed nature of dinoflagellate chromosomes (Gautier et al., 1986) raises questions concerning how they transcribe their genomes. Our high-resolution 3D visualization of the nucleus allowed us to notice the presence of a filamentous structure originating from small chromosomes and extending inside the nucleolar volume. As the properties of these threads are very similar to the chromosomes they are originating from, we believe that they represent a chromatin intermediate unfolding state. Previous studies suggested that DNA structures protruding from the chromosome core have a role in RNA transcription (Rizzo, 1991; Sigee, 1983, 1984; Soyer-Gobillard et al., 1990). Such structures, originating from the chromosomes and branching towards the nucleoplasm, have been reported in various dinoflagellate species (Bhaud et al., 2000; Decelle et al., 2021; Soyer-Gobillard et al., 1990). As the nucleolus is a prominent site for ribosomal biogenesis in eukaryotes (Hadjiolov, 1980) and rRNA genes have been localized at the nucleolus interface in dinoflagellates (Géraud et al., 1991), the arrangement observed in our cell is consistent with potential intranucleolar transcriptional activity that has been previously hypothesized (Géraud et al., 1991).

This work also highlights the importance of correlating 3D light and EM data beyond targeting purposes. The subcellular precision of such correlation allowed us to assign the emission of a fluorescent signal (far red) to a specific organelle (chloroplast). Further studies, by mapping subcellular structure to their corresponding fluorescence spectra, could allow for a more comprehensive understanding of various pigmented microorganisms, which, in turn, will further facilitate their identification in environmental samples. Furthermore, association to other complementary molecular tools such as metabarcoding, genomics, transcriptomics or *in situ* hybridization could allow for a more exhaustive understanding of these marine microorganisms in culture-free systems.

Altogether, along with providing new insight into the cell biology of dinoflagellates, our study is a proof of principle for volume CLEM as a valuable tool for the ultrastructural exploration of heterogeneous environmental samples.

## MATERIALS AND METHODS

### Sample collection

Sampling of marine plankton was performed by towing a net of 5–10 µm mesh size (Aquatic Research Instruments, Hope, ID, USA) for 10 min slowly in surface waters of the Villefranche-sur-Mer bay (France). Sampling was performed on 14 September 2021 in the early morning. Samples were filtered through serial sieves (Retsch, Haan, Germany) to collect cells measuring less than 40 µm in diameter. The fraction obtained was kept in Nalgene plastic bottles and placed in a closed cooler filled with sea water to preserve them at sea temperature and in darkness until further processing. The sample was then concentrated on a 1.2 µm meshed mixed cellulose ester membrane (Merck, Darmstadt, Germany) and pelleted using centrifugation for 5 min at 1000 ***g*** and 20°C with a swinging bucket centrifuge (Eppendorf 5427R, Hamburg, Germany).

### High-pressure freezing and freeze substitution

After the collection described above, 1.2 µl of the sample pellet was loaded in a type A gold-coated copper carrier (200 µm deep and 3 mm wide, Leica Microsystems, Wetzlar, Germany) and topped with the flat side of an aluminium type B carrier (Leica Microsystems). High-pressure freezing was performed using an EM ICE high-pressure freezer (Leica Microsystems). To allow for a very rapid freezing of the sample upon collection at sea, the instrument was set up meters away from the pier at the Institut de la Mer in Villefranche-sur-Mer (France). Samples presented here were frozen within a time window of under 2 h after being collected at sea. Cryoimmobilized samples underwent freeze substitution (EM-AFS2, Leica Microsystems) following a protocol adapted from Ronchi et al. (2021). Briefly, the samples were incubated in the freeze-substitution cocktail [0.1% uranyl acetate (Agar Scientific, Stansted, UK) in dry acetone (EMS, Hatfield, PA, USA)] for 69 h at −90°C. The temperature was raised to −45°C over 15 h (3°C/h) and the samples were further incubated for 5 h at −45°C. After rinsing with acetone, the infiltration with Lowicryl HM20 (Polysciences, Warrington, PA, USA) was performed using increasing resin concentration in steps of 6 h each. During infiltration, the temperature was increased gradually to −25°C. Three infiltration steps using 100% Lowicryl were done at −25°C for 6, 17 and 10 h, respectively. Polymerization was performed using ultraviolet light at −25°C for 48 h, followed by raising the temperature to 20°C.

### Targeting strategy

In order to target the cell of interest, we generated a 3D map of the block using confocal microscopy (Ronchi et al., 2021). For this, the sample was mounted face down on a glass-bottomed dish (glass thickness 17 µm, MatTek, Ashland, MA, USA) on a drop of water. Acquisition and laser branding were done using a Zeiss LSM 780 NLO microscope equipped with a pulsed NIR laser used in two-photon microscopy and a 25×/0.8 NA multi-immersion objective (LD-LCI Plan-Apochromat, Zeiss, Jena, Germany). The following channels were acquired: two color channels detecting the autofluorescence signal of the sample, exciting autofluorescence at 488 nm and 633 nm. Together with the 488 nm excitation channel, an image of the transmitted laser light was generated using the transmission photomultiplier tube (T-PMT) detector of the microscope. Additionally, a reflection channel was recorded. For this, the main beam splitter was changed to a T80/R20 filter reflecting 80% of the incident light and transmitting 20%. The reflection of a 633 nm laser at low intensity was measured with a multi-alkali photomultiplier tube (MA-PMT) at low gain. Reflection protection for all laser lines was removed in the beam path of the microscope. The interface between water and resin was visible as bright reflection signal in this channel and could be used to determine the axial position of the autofluorescent structures within the block.

For laser branding, the bleaching functionality of the microscope was used, with which specific regions within an image can be selectively illuminated. For these regions, the NIR laser was set to a wavelength of 850 nm. Laser power was tuned to achieve efficient branding while avoiding blebbing of the resin. With our system, we achieved this at values around 12% of the maximum power.

### Sample mounting and FIB-SEM acquisition

The block was cut parallelly to its surface in order to be 2–3 mm high, and mounted on an SEM stub (Agar Scientific) using a 1:1 mix of superglue (Loctite precision max, Henkel Corp., Rocky Hill, CT, USA) and silver paint (EM-Tec AG44, Micro to Nano, Haarlem, the Netherlands). Silver paint was further added around the block surface. The sample underwent gold sputtering for 180 s at 30 mA (Q150RS, Quorum, Laughton, UK) before insertion in the FIB-SEM chamber. FIB-SEM imaging was performed using a Zeiss Crossbeam 550, following the Atlas 3D nanotomography workflow. FIB milling was performed at 1.5 nA. SEM imaging was done with an acceleration voltage of 1.5 kV and a current of 750 pA using an energy-selective backscattered (ESB) detector (ESB grid 1100 V). Imaging of the planktonic cell was done using an 8 nm isotropic voxel size with a dwell time of 9 µs. Post-acquisition dataset alignment was performed using the automated Alignment to Median Smoothed Template (AMST) procedure from Hennies et al. (2020).

### Volume analysis and quantification

Overlay of EM and light microscopy data (Fig. 3) and most of the segmentations (Fig. 5A,B,D,E) were done using Amira software (Thermo Fisher Scientific, Waltham, MA, USA). The segmentation of the theca, nucleolus, chromosomes, starch, mitochondrion, mucocysts, trichocysts, eyespot and flagellar apparatus were done using thresholding and interpolation tools. The result of this semi-automated procedure was further manually checked. The nucleus (nuclear envelope) was manually segmented using interpolation. The segmentation of the chloroplast was performed using Microscopy Image Browser (Belevich et al., 2016) by manual annotation and interpolation (Fig. 4C). In total, the segmentation of the various organelles took 72 h. Volume quantifications were performed on segmented organelles using the Amira label analysis tool. Lengths of trichocysts were measured using the Amira measurement tool.

### SEM

Part of the sample collected as described above was fixed with 2% paraformaldehyde (Electron Microscopy Sciences) and 0.5% glutaraldehyde (Electron Microscopy Sciences) in 0.1 M marPHEM (0.1 M PHEM with the addition of 9% sucrose) (Montanaro et al., 2016) for 6 h at 4°C. The sample was then transferred to 0.1 M PHEM (60 mM PIPES, 25 mM HEPES, 2 mM MgCl_2_, 10 mM EGTA, pH 6.9) containing 1% paraformaldehyde and preserved at 4°C until further processing. The sample was then rinsed once using 0.1 M PHEM at 4°C. The sample was then dehydrated at 4°C using the following (v/v) acetone/water series: 30%, 50%, 70%, 80%, 90%, followed by two pure acetone steps. Samples were left to sediment for a duration of 3 to 12 h before each exchange to avoid loss of material. The sample was then critically point dried (CPD; CPD300, Leica Microsystems) in small containers (1–1.6 µm pore size, Vitrapore ROBU, Hattert, Germany). In the CPD program, 30 slow exchange steps were used. CPD plankton were then distributed on carbon tape placed on an SEM stub (Agar Scientific) before further gold sputtering (Quorum, Q150RS). SEM imaging was performed using a Zeiss Crossbeam 540 with an acceleration voltage of 1.5 kV and a current of 700 pA and a secondary electron secondary ion (SESI) detector.

### Dataset visualization using MoBIE

The Fiji plugin MoBIE (Pape et al., 2022 preprint) can be used to explore the different datasets. Instructions for plugin download and installation can be found using the following link: https://github.com/mobie/mobie-viewer-fiji. The data are visualized by selecting the Fiji plugin→‘MoBIE’ →‘Open MoBIE Project’ and providing the project location (https://github.com/mobie/environmental-dinoflagellate-vCLEM). The project contains the FIB-SEM dataset (‘photosynthetic dinoflagellate’) and associated segmentations, registered with the confocal stack of the full block (‘LM_fullblock’), as well as the higher-resolution confocal stack of the cell before and after trimming and branding (‘LM_pre-trim’ and ‘LM_trimmed-branded’).

## Supplementary Material

10.1242/joces.261355_sup1 Supplementary information
