# Supplementary material for: Targeted volume correlative light and electron microscopy of an environmental marine microorganism
Source: J Cell Sci. 2023 Aug 9;136(15):jcs261355. doi: 10.1242/jcs.261355 (PMC10445747; doi:10.1242/jcs.261355)
Supplement: Supplementary file 1 [file joces-136-261355_review_history.pdf]

Targeted volume correlative light and electron microscopy of an environmental marine microorganism

Karel Mocaer, Giulia Mizzon, Manuel Gunkel, Aliaksandr Halavatyi, Anna Steyer, Viola Oorschot, Martin Schorb, Charlotte Le Kieffre, Daniel P. Yee, Fabien Chevalier, Benoit Gallet, Johan Decelle, Yannick Schwab and Paolo Ronchi
DOI: 10.1242/jcs.261355

Editor: Michael Way

Review timeline

Submission to Review Commons:	30 January 2023
Submission to Journal of Cell Science:	22 May 2023
Editorial decision:	25 May 2023
First revision received:	30 June 2023
Editorial decision:	6 July 2023
Accepted:	6 July 2023

Reviewer 1

Evidence, reproducibility and clarity

I am not an expert in algal ultrastructure or TEM, and I principally found this manuscript informative and well-written. I can only make minor recommendations, although I urge the Editors to supplement this review with others from specialists within the field.

To date, to my knowledge, the Pentapharsodinium chloroplast has not been characterised at a molecular level. The placement of the nuclear lineage within the Peridiniales as a close relative or Durinskia spp. and other dinoflagellates with diatom endosymbionts raises the question of whether Pentapharsodinium possesses a peridinin-containing chloroplast, per most other dinoflagellates, or possesses a chloroplast of an alternative endosymbiotic derivation, although I suppose the single chloroplast would be typical for peridinin-containing chloroplasts from the Peridiniales. Can the authors make any inference on this from their data?

While I appreciate that this is a study of a single cell only, I would prefer some more extensive evidence that the partial chromosome unfolding identified correlates to transcriptional activity. The nucleolus is surrounded by a layer of heterochromatin and perhaps the filamentous structures involved are transcriptionally quiescent. Were the authors able to take any preliminary images of cells harvested mid-day or exposed to higher light intensities, and do they see greater chromatin unfolding in this case? Similarly I would be curious if cells visualised later in the day possess multiple rather than single chloroplasts.

Finally, I have a few (very) small grammatical corrections:

Line 39: should be "dinoflagellate biology"

Line 131: "a far red signal"

Line 196: should be "number of chloroplasts"

Line 200: by curiosity, how does the measured chloroplast volume compare to those computed in vEM studies of Symbiodinium (c.f., Uwizeye 2021)

© 2023. Published by The Company of Biologists under the terms of the Creative Commons Attribution License (https://creativecommons.org/licenses/by/4.0/).

Line 221: how does the number of observed chromosomes compare to estimated chromosome numbers in dinoflagellates from karyotyping or whole-genome sequencing?

Line 240: does the eyespot show any proximity to the mitochondria, as per the hybrid chloroplast/ mitochondrial-derived eyespots found in Warnowiacean dinoflagellates?

Significance

The application of vEM to environmental algal samples has to my knowledge not been attempted previously. If these approaches could be scaled up to a multi-cell approach and is not completely destructive to the cells, it could provide a fascinating way to connect algal morphology in the wild to other culture-free methods to understand algal biology (e.g., meta- barcoding).

Reviewer 2

Evidence, reproducibility and clarity

This is avery important and interesting contribution to the ultrastructural analysis of one determoned species among many others living together in a rich sample as a marine microplankton. In addition the work also shows the possibility to obtain fundamental volumetric informations on the various structures and organelles. The work was well planned and executed and certainly represents a tremendous effort of the members of the group. It is clearly written, explaining each detail of the methodology necessary to the understanding of the whole work.

Significance

This a phantatis piece of scientiic work where the authros were able to use the moderns three dimensional reconstruction technique possible using high resolution scanning electron to reconstruct one specific cell in a large population of heterogenous cells. The identification of one specific cell based on fluorescence images detected in a light microscopy was very important to present one new methodology to observed such types of cells. In addition to a detailed description of the strucutres and organelles found they were able to determne the area/volume occupies by each of them in cells.
Therefore I strong recommend the acceptance of the manuscript as it is.

Reviewer 3

Evidence, reproducibility and clarity

Summary

The authors present a workflow to characterize a microorganism, dinoflagellate cell, from environmental samples by vEM. The workflow enables the identification of specific taxa of interest in a heterogenous environmental sample and allows correlative fluorescence and FIB-SEM acquisition. I see no major flaws with this study. The authors present a proof of principle pipeline for utilizing CLEM to explore the ultrastructure of microorganisms.

Major comments

What is not clear, is how highly reproducible this workflow is. For example, what is the time required for each sample? How manual vs automated is each step? While, the workflow is important and sound, it would be helpful for the reader to understand a little more about the variability and throughput. It doesn't need to be exhaustive, but further characterization of the workflow would significantly improve the impact of the manuscript and should be included.

As a reader, it would be tremendously helpful to clearly state what is part of the preparation/imaging workflow and what is an application example of the workflow. If it is

© 2023. Published by The Company of Biologists under the terms of the Creative Commons Attribution License (https://creativecommons.org/licenses/by/4.0/).

intended that the post-processing is also part of the workflow, there should be significantly more details provided into the segmentation and analyses processes.

Minor comments

As this study describes a workflow that was developed for identification and imaging of microorganisms, it would be highly beneficial to the reader to have a figure that shows each of the steps, end to end.

It would also be helpful to add annotation labels and a scale bar to supplemental video 2.

Do the nonphotosynthetic species also have nuclear autofluorescence or is this just a trait of a subset of the photosynthetic species?

Significance

General assessment

The authors present an important, yet missing, workflow for characterizing microorganisms from environmental samples using vEM and CLEM. A strength of the study is that it enables identification and selection of taxa in heterogenous samples. Using correlative, confocal imaging of both auto-fluorescence and transmitted light, the authors show that specific taxa can be identified and selected for according to the organism's photosynthetic and morphological properties. To improve upon this, it could be useful for the authors to provide a list or table of potential organisms that could be selected for in this manner to exemplify the use cases of this protocol.

Advance

This study, while a proof of concept, is also one of the first examples of using vEM on environmental studies. The author's also present the potential value add of using CLEM, not just for selection purposes, but also more comprehensive identification and mapping of subcellular structures. While the workflow itself is incremental (and important), the application is quite novel.

Audience

Because the authors' study combines a vEM workflow and microorganism characterization, the potential audience is broad reaching. The workflow itself could be adapted to many different systems - beyond microorganisms - making it of use to several biological fields.

Expertise

vEM, CLEM, FIB-SEM, membrane trafficking, cell biology, tissue cell biology, machine learning

---

Author response to reviewers' comments

[The "revision plan" should delineate the revisions that authors intend to carry out in response to the points raised by the referees. It also provides the authors with the opportunity to explain their view of the paper and of the referee reports.

The document is important for the editors of affiliate journals when they make a first decision on the transferred manuscript. It will also be useful to readers of the reprint and help them to obtain a balanced view of the paper.

© 2023. Published by The Company of Biologists under the terms of the Creative Commons Attribution License (https://creativecommons.org/licenses/by/4.0/).

If you wish to submit a full revision, please use our "Full Revision" template. ***It is important to use the appropriate template to clearly inform the editors of your intentions.]***

1. General Statements [optional]

This section is optional. Insert here any general statements you wish to make about the goal of the study or about the reviews.

We would like to thank the reviewers for the time and attention they gave to our study. We really appreciate their valuable feedback and suggestions that we will implement in our revised manuscript.

Our paper is a proof of concept for a volume correlative light and electron microscopy approach to target microorganisms from environmental samples. We are planning to take into serious consideration all the suggestions from the reviewers, and address each of them as much as possible.

As this study was performed on one cell and finding more individuals would be complex due to the low abundance of this species within the analyzed sample, we don't feel comfortable inferring too strong interpretation of the biology.

Within the scope of this revision, we plan to focus especially on the methodological/technical suggestions that were made.

2. Description of the planned revisions

Insert here a point-by-point reply that explains what revisions, additional experimentations and analyses are planned to address the points raised by the referees.

Reviewer 1:

I am not an expert in algal ultrastructure or TEM, and I principally found this manuscript informative and well-written. I can only make minor recommendations, although I urge the Editors to supplement this review with others from specialists within the field.

We thank the reviewer for their positive comment on the manuscript as well as for the interesting following discussion points.

To date, to my knowledge, the Pentapharsodinium chloroplast has not been characterised at a molecular level. The placement of the nuclear lineage within the Peridiniales as a close relative or Durinskia spp. and other dinoflagellates with diatom endosymbionts raises the question of **whether Pentapharsodinium possesses a peridinin-containing chloroplast, per most other dinoflagellates, or possesses a chloroplast of an alternative endosymbiotic derivation**, although I suppose the single chloroplast would be typical for peridinin-containing chloroplasts from the Peridiniales. **Can the authors make any inference on this from their data?**

In the course of this study, we did not perform molecular characterization of the chloroplast. Therefore, formal answer to this question would not be possible based on our data.

However, we have evidence that suggest that the chloroplast from *P. tyrrhenica* does not come here from a diatom endosymbiont.

1/ In the suggested cases of diatom endosymbiont origin of the chloroplast, eg in the genus *Durinskia, Yamada et al., 2019* show that *Durinskia capensis* or *Durinskia kwazulunatalensis* can present diatom organelles such as chloroplast, mitochondria, nucleus, or their remnants for a certain amount of time after uptake. The detailed analysis of our vEM data does not show these diatom organelles, thus ruling out the hypothesis.

© 2023. Published by The Company of Biologists under the terms of the Creative Commons Attribution License (https://creativecommons.org/licenses/by/4.0/).

2/ Additionally, the eyespot of dinoflagellate species possessing diatom association seems to be characteristic to these cells (*Horiguchi., 2007, Hoppenrath et al., 2017*). In our case, the eyespot of *P. tyrrhenica* corresponds to a distinct type (type IA, thought to be in peridinin dino-chloroplast according to *Hoppenrath., 2017*), that varies from the one observed in *Durinksia baltica*, or other dinoflagellates species possessing these diatom associations (*Horiguchi., 2007, Hoppenrath et al., 2017*).

Therefore, the chloroplast of our cell does not seem to be acquired from a diatom. However, as we don't have molecular evidence at this stage, and are not able to perform such analyses, we prefer not to address this question in the manuscript.

While I appreciate that this is a study of a single cell only, I would prefer some more extensive **evidence that the partial chromosome unfolding identified correlates to transcriptional activity**. The nucleolus is surrounded by a layer of heterochromatin and perhaps the filamentous structures involved are transcriptionally quiescent. **Were the authors able to take any preliminary images of cells harvested mid-day or exposed to higher light intensities, and do they see greater chromatin unfolding in this case? Similarly I would be curious if cells visualised later in the day possess multiple rather than single chloroplasts.**

Our description is here based on one microorganism of very low abundance, and we do not have data for it across conditions. Therefore, we would not be comfortable inferring too many details in this paper and will thus modify the text in order be more careful about the potential role of these fibres and their putative association to transcription. We are planning to address this interesting point in the future, in a more biologically focused paper in preparation. Nevertheless, similar structures have been described protruding in the nucleoplasm in other species (*Soyer., 1981, Bhaud et al., 2000, Decelle et al., 2021*) and have been suggested to be associated to RNA transcription (*Sigee., 1983*). We will make sure to cite and discuss more literature on this point.

Line 39: should be "dinoflagellate biology"
Line 131: "a far red signal"
Line 196: should be "number of chloroplasts"

We thank the reviewer, the errors will be corrected in the revised version.

Line 200: by curiosity, how does the measured chloroplast volume compare to those computed in vEM studies of Symbiodinium (c.f., Uwizeye 2021)

The measured chloroplast volume in our cell differs to those computed in Symbiodinium in *Uwizeye et al., 2021*. Indeed, in *Uwizeye et al., 2021*, the chloroplast represents 30% of the cell volume. This is more than in our cell for which the chloroplast represents only 9.5% of the cell volume. However, these two cells are different species and come from different growth conditions (culture VS environment). These factors potentially contribute to morphometric variations as the environment possesses different types and amounts of nutrients and light resources.

Line 221: how does the number of observed chromosomes compare to estimated chromosome numbers in dinoflagellates from karyotyping or whole-genome sequencing?

To our knowledge, there is a wide variability in terms of number of chromosomes observed between dinoflagellate species. *Bhaud et al (2000)* reports the presence of between 4 and 200 chromosomes depending on the species. Unfortunately, we did not find information concerning the number of chromosomes for this species to compare with our analysis. However, we are planning in the revised version to discuss how our workflow is complementary to other methods such as genomics, transcriptomics and light microscopy towards better understanding of marine organism.

Line 240: does the eyespot show any proximity to the mitochondria, as per the hybrid chloroplast/mitochondrial-derived eyespots found in Warnowiacean dinoflagellates?

© 2023. Published by The Company of Biologists under the terms of the Creative Commons Attribution License (https://creativecommons.org/licenses/by/4.0/).

In our study the eyespot is composed of pigment globules, organized as a sheet, located inside the chloroplast and facing towards the theca. This type of arrangement seems to be distinct to the chloroplast/mitochondria-derived eyespot described in *Warnowiaceae* (*Colley and Nilson., 2016*). Additionally, for the revision of this paper we will further segment the mitochondria to investigate any potential proximity to the eyespot. We will also put the eyespot of *P. tyrrhenica* in context with other types described in the literature.

Reviewer #1 (Significance (Required)):

The application of vEM to environmental algal samples has to my knowledge not been attempted previously. If these approaches could be scaled up to a multi-cell approach and is not completely destructive to the cells, it could provide a fascinating way to connect algal morphology in the wild to other culture-free methods to understand algal biology (e.g., meta-barcoding).

We would like to address the interesting comment concerning the possibility to combine vEM along with other molecular tools to study organisms from a culture free method. While FIB-SEM is a destructive method, it could be used in combination with other methods and thus synergize towards the characterization of the environmental samples. We will add a few sentences in the discussion, as a forward look, as it represents indeed an important axis of our future research, where molecular taxonomy (e.g. metabarcoding) will be correlated to morphological characterisations. We believe such approaches will be useful beyond the study of micro-algae, and will make this point clear in the discussion.

Reviewer #2 (Evidence, reproducibility and clarity (Required)):

This is avery important and interesting contribution to the ultrastructural analysis of one scientific species among many others living together in a rich sample as a marine microplankton. In addition the work also shows the possibility to obtain fundamental volumetric informations on the various structures and organelles. The work was well planned and executed and certainly represents a tremendous effort of the members of the group. It is clearly written, explaining each detail of the methodology necessary to the understanding of the whole work.

Reviewer #2 (Significance (Required)):

This a phantatis piece of scientific work where the authros were able to use the moderns three dimensional reconstruction technique possible using high resolution scanning electron to reconstruct one specific cell in a large population of heterogenous cells. The identification of one specific cell based on fluorescence images detected in a light microscopy was very important to present one new methodology to observed such types of cells. In addition to a detailed description of the strucutres and organelles found they were able to determne the area/volume occupies by each of them in cells.

Therefore I strong recommend the acceptance of the manuscript as it is.

We thank the reviewer for their appreciation of our work.

Reviewer #3 (Evidence, reproducibility and clarity (Required)):

Summary --

The authors present a workflow to characterize a microorganism, dinoflagellate cell, from environmental samples by vEM. The workflow enables the identification of specific taxa of interest in a heterogenous environmental sample and allows correlative fluorescence and FIB-SEM acquisition. I see no major flaws with this study. The authors present a proof of principle pipeline for utilizing CLEM to explore the ultrastructure of microorganisms.

© 2023. Published by The Company of Biologists under the terms of the Creative Commons Attribution License (https://creativecommons.org/licenses/by/4.0/).

Major comments --

What is not clear, is how highly reproducible this workflow is. For example, **what is the time required for each sample? How manual vs automated is each step?** While, the workflow is important and sound, it would be helpful for the reader to understand a little more about the **variability and throughput**. It doesn't need to be exhaustive, but further characterization of the workflow would significantly improve the impact of the manuscript and should be included. As a reader, it would be tremendously helpful to clearly state what is part of the preparation/imaging workflow and what is an application example of the workflow. If it is intended that the post- processing is also part of the workflow, there should be significantly more details provided into the segmentation and analyses processes.

We thank the reviewers for pointing this out. This workflow has been applied to several blocks and repeated imaging of a subset of species could be achieved with 100% success. We will show this in a follow up paper. Moreover, the same method has been applied for targeted FIB-SEM acquisition of samples expressing fluorescent proteins (*Ronchi et al., 2021*). Therefore, we believe our workflow is highly reproducible and robust. We are planning to address the concern on technical variability in our revised version, together with a better explanation of time scales and automated VS manual aspect of the steps that are used for this workflow. We will also add information concerning the segmentation.

Minor comments --

As this study describes a workflow that was developed for identification and imaging of microorganisms, it would be highly beneficial to the reader to have a figure that shows each of the steps, end to end.

We thank the reviewer for this suggestion. We plan to add a supplementary figure that describes the workflow step by step.

It would also be helpful to add annotation labels and a scale bar to supplemental video 2.

We thank the reviewer for spotting this missing information. We plan to add annotation labels as well as a scale bar to the video S2.

Do the nonphotosynthetic species also have nuclear autofluorescence or is this just a trait of a subset of the photosynthetic species?

We thank the reviewer for this question. From our analysis, we can’t conclude that it is a trait of some photosynthetic species compared to non-photosynthetic ones. However, heterogeneous autofluorescence profiles, even though unexplained, represent a valuable tool to discriminate between cell types. We would like to further address this point in the manuscript that such fluorescence profiles, even though unexplained, can contribute to the identification of specific cell types.

Reviewer #3 (Significance (Required)):

General assessment --

The authors present an important, yet missing, workflow for characterizing microorganisms from environmental samples using vEM and CLEM. A strength of the study is that it enables identification and selection of taxa in heterogenous samples. Using correlative, confocal imaging of both auto-fluorescence and transmitted light, the authors show that specific taxa can be identified and selected for according to the organism's photosynthetic and morphological properties.

We thank the reviewer for their positive comments.

© 2023. Published by The Company of Biologists under the terms of the Creative Commons Attribution License (https://creativecommons.org/licenses/by/4.0/).

To improve upon this, it could be useful for the authors to provide a list or table of potential organisms that could be selected for in this manner to exemplify the use cases of this protocol.

We thank the reviewer for their suggestion. We plan to add a supplementary figure with a gallery of different cells and their identification.

Advance --

This study, while a proof of concept, is also one of the first examples of using vEM on environmental studies. The author's also present the potential value add of using CLEM, not just for selection purposes, but also more comprehensive identification and mapping of subcellular structures. While the workflow itself is incremental (and important), the application is quite novel.

Audience --

Because the authors' study combines a vEM workflow and microorganism characterization, the potential audience is broad reaching. The workflow itself could be adapted to many different systems - beyond microorganisms - making it of use to several biological fields.

We thank the reviewer for their positive comments.

Expertise --

vEM, CLEM, FIB-SEM, membrane trafficking, cell biology, tissue cell biology, machine learning

3. Description of the revisions that have already been incorporated in the transferred manuscript

Please insert a point-by-point reply describing the revisions that were already carried out and included in the transferred manuscript. If no revisions have been carried out yet, please leave this section empty.

4. Description of analyses that authors prefer not to carry out

Please include a point-by-point response explaining why some of the requested data or additional analyses might not be necessary or cannot be provided within the scope of a revision. This can be due to time or resource limitations or in case of disagreement about the necessity of such additional data given the scope of the study. Please leave empty if not applicable.

First decision letter

MS ID#: JOCES/2023/261355
MS TITLE: Targeted volume Correlative Light and Electron Microscopy of an environmental marine microorganism
AUTHORS: Karl Mocaer, Giulia Mizzon, Manuel Gunkel, Aliaksandr Halavatyi, Anna Maria Steyer, Viola Oorschot, Martin Schorb, Charlotte Le Kieffre, Daniel P Yee, Fabien Chevalier, Benoit Gallet, Johan Decelle, Yannick Schwab, and Paolo Ronchi
ARTICLE TYPE: Research Article

We have now reached a decision on the above manuscript.

To see the reviewers' reports and a copy of this decision letter, please go to: https://submit-jcs.biologists.org and click on the 'Manuscripts with Decisions' queue in the Author Area. (Corresponding author only has access to reviews.)

© 2023. Published by The Company of Biologists under the terms of the Creative Commons Attribution License (https://creativecommons.org/licenses/by/4.0/).

Based on the reviews from review commons and your response to their comments I would be happy to see a revised MS along the lines you have indicated.

Please ensure that you clearly highlight all changes made in the revised manuscript. Please avoid using 'Tracked changes' in Word files as these are lost in PDF conversion.

I should be grateful if you would also provide a point-by-point response detailing how you have dealt with the points raised by the reviewers in the 'Response to Reviewers' box. Please attend to all of the reviewers' comments. If you do not agree with any of their criticisms or suggestions please explain clearly why this is so.

First revision

Author response to reviewers' comments

Response to reviewers

We would like to thank the reviewers for the time and attention they gave to our study. We really appreciate their valuable feedback and suggestions that we implemented in our revised manuscript. All changes from the original manuscript are highlighted in yellow.

Reviewer #1

Reviewer #1 (Evidence, reproducibility and clarity (Required)):

I am not an expert in algal ultrastructure or TEM, and I principally found this manuscript informative and well-written. I can only make minor recommendations, although I urge the Editors to supplement this review with others from specialists within the field.

We thank the reviewer for the positive comment on the manuscript as well as for the interesting following discussion points.

To date, to my knowledge, the Pentapharsodinium chloroplast has not been characterised at a molecular level. The placement of the nuclear lineage within the Peridiniales as a close relative or Durinskia spp. and other dinoflagellates with diatom endosymbionts raises the question of whether Pentapharsodinium possesses a peridinin-containing chloroplast, per most other dinoflagellates, or possesses a chloroplast of an alternative endosymbiotic derivation, although I suppose the single chloroplast would be typical for peridinin-containing chloroplasts from the Peridiniales. Can the authors make any inference on this from their data?

In the course of this study, we did not perform molecular characterization of the chloroplast. Therefore, formal answer to this question would not be possible based on our data.
However, we have evidence that suggest that the chloroplast from *P. tyrrhenica* does not come here from a diatom endosymbiont.

1/ In the suggested cases of diatom endosymbiont origin of the chloroplast, eg in the genus *Durinskia, Yamada et al., 2019* show that *Durinskia capensis* or *Durinskia kwazulunatalensis* can present diatom organelles such as chloroplast, mitochondria, nucleus, or their remnants for a certain amount of time after uptake. The detailed analysis of our vEM data does not show these diatom organelles, thus ruling out the hypothesis.

2/ Additionally, the eyespot of dinoflagellate species possessing diatom association seems to be characteristic to these cells (*Horiguchi., 2007, Hoppenrath et al., 2017*). In our case, the eyespot of *P. tyrrhenica* corresponds to a distinct type (type IA, thought to be in peridinin dino-chloroplast according to *Hoppenrath., 2017*), that varies from the one observed in *Durinksia baltica*, or other

© 2023. Published by The Company of Biologists under the terms of the Creative Commons Attribution License (https://creativecommons.org/licenses/by/4.0/).

dinoflagellates species possessing these diatom associations (*Horiguchi., 2007, Hoppenrath et al., 2017*).

Therefore, the chloroplast of our cell does not seem to be acquired from a diatom. However, as we don't have molecular evidence at this stage, and this is not the main scope of our manuscript, we prefer not to address this question.

While I appreciate that this is a study of a single cell only, I would prefer some more extensive evidence that the partial chromosome unfolding identified correlates to transcriptional activity. The nucleolus is surrounded by a layer of heterochromatin and perhaps the filamentous structures involved are transcriptionally quiescent. Were the authors able to take any preliminary images of cells harvested mid-day or exposed to higher light intensities, and do they see greater chromatin unfolding in this case? Similarly I would be curious if cells visualised later in the day possess multiple rather than single chloroplasts.

Our description is here based on one microorganism of very low abundance, and we do not have data for it across conditions. Therefore, we are not able to address potential circadian variation of chromatin unfolding and chloroplast number.
However, in the revised manuscript we have discussed the link between the observed chromatin unfolding and transcriptional activity, based on existing literature. Indeed, similar structures have been described protruding in the nucleoplasm in other species (*Soyer., 1981, Bhaud et al., 2000, Decelle et al., 2021*) and have been suggested to be associated to RNA transcription (*Sigee., 1983*). This point is addressed in line 364-373:

Previous studies suggested that DNA structures protruding from the chromosome core have a role in RNA transcription (Rizzo, 1991; Sigee, 1983; Sigee, 1984; Soyer-Gobillard et al., 1990). Such structures, originating from the chromosomes and branching towards the nucleoplasm, have been reported in various dinoflagellate species (Bhaud et al., 2000; Decelle et al., 2021; Soyer-Gobillard et al., 1990). As the nucleolus is a prominent site for ribosomal biogenesis in eukaryotes (Hadjiolov, 1980) and rRNA genes have been localized at the nucleolus interphase in dinoflagellates (Géraud et al., 1991), the arrangement observed in our cell is consistent with potential intranucleolar transcriptional activity which has been previously hypothesized (Géraud et al., 1991).

As we felt that our statement in the abstract on this topic may have been too strong, we rephrased it (line 50-51): Moreover, we observed partial chromatin unfolding, potentially associated with transcription activity in these organisms where chromosomes are permanently condensed.

Finally, I have a few (very) small grammatical corrections: We thank the reviewer for spotting these mistakes.
Line 39: should be "dinoflagellate biology"
Corrected (line 52)
Line 131: "a far red signal"
Corrected (line 162)
Line 196: should be "number of chloroplasts" Corrected (line 234)

Line 200: by curiosity, how does the measured chloroplast volume compare to those computed in vEM studies of Symbiodinium (c.f., Uwizeye 2021)

The measured chloroplast volume in our cell differs to those computed in Symbiodinium in *Uwizeye et al., 2021*. Indeed, in *Uwizeye et al., 2021*, the chloroplast represents 30% of the cell volume. This is more than in our cell for which the chloroplast represents only 9.5% of the cell volume. However, these two cells are different species and come from different growth conditions (culture VS environment). These factors potentially contribute to morphometric variations as the environment possesses different types and amounts of nutrients and light resources. We do not feel this needs to be addressed in the text.

Line 221: how does the number of observed chromosomes compare to estimated chromosome numbers in dinoflagellates from karyotyping or whole-genome sequencing?

© 2023. Published by The Company of Biologists under the terms of the Creative Commons Attribution License (https://creativecommons.org/licenses/by/4.0/).

To our knowledge, there is a wide variability in terms of number of chromosomes observed between dinoflagellate species. *Bhaud et al (2000)* reports the presence of between 4 and 200 chromosomes depending on the species. Unfortunately, we have not found information concerning the number of chromosomes for *E. Tyrrhenica* to compare with our analysis. However, as we feel that it is important to mention complementary approaches such as genomics, transcriptomics and light microscopy, which contribute to a better understanding of environmemntal organisms, we have discussed this in the revised manuscript (lines 380-383):

Furthermore, association to other complementary molecular tools such as metabarcoding, genomics, transcriptomics, or *in situ* hybridization, could allow for a more exhaustive understanding of these marine microorganisms in culture free systems.

Line 240: does the eyespot show any proximity to the mitochondria, as per the hybrid chloroplast/ mitochondrial-derived eyespots found in Warnowiacean dinoflagellates?

In our study the eyespot is composed of pigment globules, organized as a sheet, located inside the chloroplast and facing towards the theca. This type of arrangement seems to be distinct to the chloroplast/mitochondria-derived eyespot described in *Warnowiaceae* (*Colley and Nilson., 2016*). Additionally, in order to further address this point, we have segmented and further analysed the mitochondrial volume. This organelle is not closely associated to the eyespot structure, therefore confirming the different nature of the eyespot, rather corresponding to the type 1A (according to the classification of Hoppenrath et al., 2017). The new data about the mitochondria are presented in Fig. 5D and video S2. Discussion about the eyespot type has been added to the manuscript (line 285-288):

Based on this arrangement, we believe it belongs to the category I(A) of the eyespot classification (Hoppenrath, 2017), which has previously been described for other organisms from the family of Peridiniaceae (Calado et al., 1999; Messer and Ben-Shaul, 1969; Moestrup and Daugbjerg, 2007).

Reviewer #1 (Significance (Required)):

The application of vEM to environmental algal samples has to my knowledge not been attempted previously. If these approaches could be scaled up to a multi-cell approach and is not completely destructive to the cells, it could provide a fascinating way to connect algal morphology in the wild to other culture-free methods to understand algal biology (e.g., meta-barcoding).

While FIB-SEM is a destructive and time-consuming method, workflows like the one presented here, due to a precise targeted acquisition, allow to gain in acquisition efficiency. The identification of subcellular structures due to the autofluorescence profile of the cell could help to further restrict the imaged area and therefore potentially scale up this type of analysis. This is now emphasised in the discussion (line 332-337):

In the future, using this dataset as a reference and taking advantage of specific autofluorescence signatures, it may be possible to further restrict the vEM acquisition to a subcellular volume to answer specific biological questions. Overall, such an optimization of the acquisition time can then allow to scale up vEM analyses. Segmentation is another crucial, but time-consuming part of the workflow. However, with experience the segmentation can be significantly accelerated. The almost entirely manual approach we used for this study (See Material and Method) took a trained user approximately 72 h. We envision that artificial intelligence can contribute to automate this step, therefore removing another bottleneck towards scaling up of the method (Heinrich et al., 2021).

Moreover, vEM could be used in parallel to other methods and thus synergize towards the characterization of the environmental samples. We added a sentence in the discussion, as a forward look, as it represents indeed an important axis of our future research, where molecular taxonomy (e.g. metabarcoding) will be correlated to morphological characterisations (lines 380-383):

© 2023. Published by The Company of Biologists under the terms of the Creative Commons Attribution License (https://creativecommons.org/licenses/by/4.0/).

Furthermore, association to other complementary molecular tools such as metabarcoding, genomics, transcriptomics, or *in situ* hybridization, could allow for a more exhaustive understanding of these marine microorganisms in culture free systems.

We believe such approaches will be useful beyond the study of micro-algae, as we suggested at the end of the discussion (lines 368-370).

Reviewer #2

Reviewer #2 (Evidence, reproducibility and clarity (Required)):

This is avery important and interesting contribution to the ultrastructural analysis of one determoned species among many others living together in a rich sample as a marine microplankton. In addition the work also shows the possibility to obtain fundamental volumetric informations on the various structures and organelles. The work was well planned and executed and certainly represents a tremendous effort of the members of the group. It is clearly written, explaining each detail of the methodology necessary to the understanding of the whole work.

Reviewer #2 (Significance (Required)):

This a phantatis piece of scientiic work where the authros were able to use the moderns three dimensional reconstruction technique possible using high resolution scanning electron to reconstruct one specific cell in a large population of heterogenous cells. The identification of one specific cell based on fluorescence images detected in a light microscopy was very important to present one new methodology to observed such types of cells. In addition to a detailed description of the strucutres and organelles found they were able to determne the area/volume occupies by each of them in cells. Therefore I strong recommend the acceptance of the manuscript as it is.

We thank the reviewer for the positive feedback and appreciation of our work.

Reviewer #3

Reviewer #3 (Evidence, reproducibility and clarity (Required)):

Summary --

The authors present a workflow to characterize a microorganism, dinoflagellate cell, from environmental samples by vEM. The workflow enables the identification of specific taxa of interest in a heterogenous environmental sample and allows correlative fluorescence and FIB-SEM acquisition. I see no major flaws with this study. The authors present a proof of principle pipeline for utilizing CLEM to explore the ultrastructure of microorganisms.

Major comments --

What is not clear, is how highly reproducible this workflow is. For example, what is the time required for each sample? How manual vs automated is each step? While, the workflow is important and sound, it would be helpful for the reader to understand a little more about the variability and throughput. It doesn't need to be exhaustive, but further characterization of the workflow would significantly improve the impact of the manuscript and should be included.

We thank the reviewers for pointing this out. This workflow has been applied to several blocks and repeated imaging of a subset of species could be achieved with 100% success. We will show this in a follow up paper. Moreover, the same method has been applied for targeted FIB-SEM acquisition of samples expressing fluorescent proteins (*Ronchi et al., 2021*) with a similar success rate. Therefore, we believe our workflow is highly reproducible and robust. We have added a sentence in the discussion (lines 315-318):

© 2023. Published by The Company of Biologists under the terms of the Creative Commons Attribution License (https://creativecommons.org/licenses/by/4.0/).

A similar workflow has been shown to work for exogenously expressed fluorescent proteins (Ronchi et al., 2021) or small molecules live dyes (Imprima et al., 2023), with a success rate close to 100%. We have now demonstrated that the same principle can be applied to endogenous fluorescence signals with similar robustness, opening the way for environmental sample analysis.

In the revised version of the manuscript, we have discussed more in details the time scale of such acquisitions and analysis:
Line 327-329 (discussion):

This allowed us to optimize the imaging time and generate the entire dataset of the cell

(~15 µm diameter) in less than 48 h. However, depending on the volume to acquire, the time for imaging will vary. For instance, some of the larger cells found in our samples (around 40µm in diameter) would be acquired in 5 days using the same imaging settings.

Line 466 (materials and methods):

In total, the segmentation of the various organelles took 72h.

In the revised version of the manuscript we have detailed in “Materials and methods” which post-acquisition processing steps could be performed automatically vs manually.
Line 455-466:
Post-acquisition dataset alignment was performed using the automated AMST procedure from Hennies et al., 2020.

Volume analysis and quantification

Overlay of EM and LM data (Fig. 3) and most of the segmentations (Fig. 4A,B,D,E)

were done using Amira (Thermo Fisher Scientific, Waltham, MA, USA). The segmentation of the theca, nucleolus, chromosomes, starch, mitochondrion, mucocysts, trichocysts, eyespot and flagellar apparatus were done using thresholding and interpolation tools. The result of this semi-automated procedure was further manually proofread. The nucleus (nuclear envelope) was manually segmented using interpolation. The segmentation of the chloroplast was performed using Microscopy Image Browser (Belevich et al, 2016) by manually annotating and interpolating (Fig. 4C). In total, the segmentation of the various organelles took 72h.

As a reader, it would be tremendously helpful to clearly state what is part of the preparation/imaging workflow and what is an application example of the workflow. If it is intended that the post-processing is also part of the workflow, there should be significantly more details provided into the segmentation and analyses processes.

To address this point, we have added a figure (fig. 1) and an introductory paragraph (lines 119-132) that outline the main steps of our workflow.

Workflow

In order to study environmental marine microorganisms, we established a new workflow (Fig. 1). It consists in collection at sea (Fig. 1A), fractionation and concentration of the sample, followed by its rapid cryo-immobilization on site (Fig. 1B, see Material and Methods). These frozen samples are then freeze substituted and resin embedded according to Ronchi et al., 2021, in order to preserve the autofluorescence properties of the cells (Fig. 1B). A 3D map of the entire block is generated by confocal microscopy, allowing us to identify a variety of microorganisms and their genus (Fig. 1C). A specific cell of interest can then be localized in x, y and in relation to the block’s surface (Fig. 1D). After trimming away the resin on top of the targeted cell, branding of a pattern around this organism (Fig. 1D) is performed to allow for its precise acquisition using FIB-SEM imaging (Fig. 1E). The dataset is then aligned, and semi-automated segmentation is done for further

© 2023. Published by The Company of Biologists under the terms of the Creative Commons Attribution License (https://creativecommons.org/licenses/by/4.0/).

analysis of lengths and volumes of various subcellular structures as well as their visualization (Fig. 1F). The single steps of this workflow will be further detailed in the next paragraphs.

Moreover, we included additional information in material and methods about the segmentation of the cell (lines 460-466):

The segmentation of the theca, nucleolus, chromosomes, starch, mitochondrion, mucocysts, trichocysts, eyespot and flagellar apparatus were done using thresholding and interpolation tools. The result of this semi-automated procedure was further manually proofread. The nucleus (nuclear envelope) was manually segmented using interpolation. The segmentation of the chloroplast was performed using Microscopy Image Browser (Belevich et al, 2016) by manually annotating and interpolating (Fig. 4C).

As this study describes a workflow that was developed for identification and imaging of microorganisms, it would be highly beneficial to the reader to have a figure that shows each of the steps, end to end.

We thank the reviewer for this suggestion. The new Figure 1 describes the main steps of the workflow.

It would also be helpful to add annotation labels and a scale bar to supplemental video 2

We thank the reviewer for this suggestion. We have added labels for the different organelles and a scale bar to video 2.

Do the nonphotosynthetic species also have nuclear autofluorescence or is this just a trait of a subset of the photosynthetic species?

We thank the reviewer for this question. From our analysis, we can't conclude that it is a trait of some photosynthetic species compared to non-photosynthetic ones. However, we believe that heterogeneous autofluorescence profiles, even though unexplained, represent a valuable tool to discriminate between cell types. We have added a sentence to highlight this (lines 194-196):

Additionally, even though unexplained, autofluorescence signal as the one emitted here by the nucleus area can contribute to a finer discrimination between cell types.

Reviewer #3 (Significance (Required)):

General assessment –

The authors present an important, yet missing, workflow for characterizing microorganisms from environmental samples using vEM and CLEM. A strength of the study is that it enables identification and selection of taxa in heterogenous samples. Using correlative, confocal imaging of both auto-fluorescence and transmitted light, the authors show that specific taxa can be identified and selected for according to the organism's photosynthetic and morphological properties.

We thank the reviewer for their positive comments.

To improve upon this, it could be useful for the authors to provide a list or table of potential organisms that could be selected for in this manner to exemplify the use cases of this protocol.

We thank the reviewer for the suggestion. Although a comprehensive list of microorganisms in the samples is not possible due to the amount, heterogeneity and complexity of the cells, we have included in the revised version a supplementary figure where we outline a larger subset of organisms that we could identify. We also added reference to this figure in the main text (line 149-153): Using excitation light at

© 2023. Published by The Company of Biologists under the terms of the Creative Commons Attribution License (https://creativecommons.org/licenses/by/4.0/).

488 nm and 633 nm along with transmitted light to visualize cell morphology, we could distinguish different genera in the block. For instance, we could observe dinoflagellates such as *Protoperidinium*, *Prorocentrum* or *Oxytoxum*, as well as other organisms like diatoms or coccolithophores (Fig. 2 B-M , Fig. S1)
.
Advance --

This study, while a proof of concept, is also one of the first examples of using vEM on environmental studies. The author's also present the potential value add of using CLEM, not just for selection purposes, but also more comprehensive identification and mapping of subcellular structures. While the workflow itself is incremental (and important), the application is quite novel.

Audience --

Because the authors' study combines a vEM workflow and microorganism characterization, the potential audience is broad reaching. The workflow itself could be adapted to many different systems - beyond microorganisms - making it of use to several biological fields.

We thank the reviewer for their positive comments.

Expertise --

vEM, CLEM, FIB-SEM, membrane trafficking, cell biology, tissue cell biology, machine learning

---

Second decision letter

MS ID#: JOCES/2023/261355
MS TITLE: Targeted volume Correlative Light and Electron Microscopy of an environmental marine microorganism
AUTHORS: Karl Mocaer, Giulia Mizzon, Manuel Gunkel, Aliaksandr Halavatyi, Anna Maria Steyer, Viola Oorschot, Martin Schorb, Charlotte Le Kieffre, Daniel P Yee, Fabien Chevalier, Benoit Gallet, Johan Decelle, Yannick Schwab, and Paolo Ronchi
ARTICLE TYPE: Research Article

Thank you for sending your manuscript to Journal of Cell Science through Review Commons.

I am happy to tell you that your manuscript has been accepted for publication in Journal of Cell Science, pending standard ethics checks.

© 2023. Published by The Company of Biologists under the terms of the Creative Commons Attribution License (https://creativecommons.org/licenses/by/4.0/).
